# Improving the Quality of Medical Education Research Through the Fundamentals of Neurology Education Research Series

**DOI:** 10.1212/NE9.0000000000200183

**Published:** 2024-12-30

**Authors:** Andres Fernandez, Roy E. Strowd

**Affiliations:** 1 Thomas Jefferson University, Philadelphia, PA; and; 2 Wake Forest University School of Medicine, Winston-Salem, NC.

High-quality medical education research is essential for advancing the training and preparation of health care professionals. As the demand for evidence-based practices in education grows, so too does the need to ensure that medical education research meets high standards of rigor and reliability. The field often faces criticism for methodological weaknesses, including limited generalizability or transferability, inadequate controls, underpowered sample sizes, and inconsistent reporting. Improving the quality of research is not merely an academic pursuit; it is essential for advancing teaching strategies, optimizing learner outcomes, and ultimately enhancing patient care.

Since its first issue in September 2022, the *Neurology*® *Education* journal has provided a venue for dissemination of education scholarship in the clinical neurosciences. The journal has become the established home for medical education research in neurology and clinical neurosciences. Numerous high-quality, practice-advancing, and evidence-informing articles have been published. At the same time, the journal has received many single-institution, methodologically flawed, and insufficiently framed studies underscoring the opportunity to elevate the quality of research being conducted in our field. Now that the initial work of starting and establishing the journal has progressed, we reflect on the question, how do we maintain the current track record of excellent publications while enhancing the ability of our readers to appraise the articles in the journal and for prospective authors to develop and conduct rigorous educational scholarship?

To answer this question, we should first recognize that the nature of educational research differs from the standard models of biomedical research. Medical education research often draws heavily on the traditions of the social sciences, incorporating both qualitative and quantitative approaches to explore complex educational phenomena. Unlike purely biomedical research, where rigor is often defined through metrics such as reproducibility and statistical significance, qualitative designs in medical education require a nuanced understanding of rigor tailored to the educational context. Educational research is also more often underpinned by theoretical frameworks and characterized by the application of qualitative methodologies and different methods of data collection (e.g., interviews or focus groups). We appreciate that our readers and prospective authors might not be fully versed in the different elements involved in rigorous educational research, and these potential gaps provide the journal an opportunity to advance its mission by developing targeted educational content through a series of articles to address these needs.

To this end, we have created the Fundamentals of Neurology Education Research (FuNER) series of articles to enhance the journal's content related to the educational research process. The FuNER series is not a single topical issue on education research but, instead, will be a recurrent series of articles on education research that is tailored to a neurology and clinical neuroscience audience. The goal is for the FuNER articles to have practical content that can be directly used to guide study planning, design, conduct, analysis, and interpretation. Each article will highlight neurology-centered educational case examples and targeted recommendations and include commonly encountered methodological gaps observed by reviewers when assessing manuscripts submitted to the journal. In addition to providing guidance to prospective authors, the topics can also be useful for readers to improve their ability to critically appraise the articles published in the journal.

The initial articles in the series will follow a sequential order, starting with topics that should be considered at the planning stages of an education research project. This includes concepts from the philosophy of science, conceptual frameworks, theoretical frameworks, and methodological choices. Subsequent focus will then shift to topics directly related to conducting a project such as different methods of data collection and data analysis approaches.

This issue of *Neurology: Education* includes the first two articles in the FuNER series. The first article on the philosophy of science discusses the concepts of ontology, epistemology, and paradigms as foundational concepts to apply when designing educational research.^1^ The second article on conceptual frameworks provides a detailed description of the process of building a conceptual framework for an educational research project.^2^ Frameworks provide a structured lens through which researchers can interpret complex phenomena and situate findings within the ongoing conversation in the literature by linking the study's findings to existing theories, models, or research. This connection not only provides context for the study but also enables researchers to build on or challenge existing knowledge, fostering a deeper and more cohesive understanding within the field. Together, these two articles provide foundational concepts and steps involved in building rigorous educational scholarship.

The FuNER series is not a static series because new topics can be added as needs are identified. We intend for the FuNER series to be a resource for neurology educators, so the ongoing input from the journal's audience will be invaluable to identify next steps and new areas. Authors interested in writing a methods paper for the series should contact the journal. We hope this series will provide a useful resource as we aim to elevate the rigor of the neurology education field through enhanced educational research knowledge and skills.
